# Younger Children with Respiratory Tract Infections Are More Exposed to Off-Label Treatments: An Exploratory Retrospective Study in a Pediatric Emergency Setting

**DOI:** 10.3390/children11060735

**Published:** 2024-06-16

**Authors:** Greta Venckute, Erika Zekaite-Vaisniene, Urte Oniunaite, Lina Jankauskaite

**Affiliations:** 1 Department of Pediatrics, Medical Academy, Lithuanian University of Health Sciences, LT-44307 Kaunas, Lithuania; greta.venskute@stud.lsmu.lt; 2 Department of Neonatology, Medical Academy, Lithuanian University of Health Sciences, LT-50161 Kaunas, Lithuania; erika.zekaite.vaisniene@kaunoklinikos.lt; 3 Faculty of Medicine, Medical Academy, Lithuanian University of Health Sciences, LT-44307 Kaunas, Lithuania; urte.oniunaite@stud.lsmu.lt

**Keywords:** emergency department, off-label drugs, pediatric, respiratory infections

## Abstract

Off-label drug use is prevalent in the pediatric population and represents a patient safety concern. We aimed to identify factors for off-label drug use in our pediatric emergency department (PED). Methods. We performed a retrospective data analysis. All patients aged 0–18 referred to PED from 1 September to 1 October 2022, were included. Further analysis was performed when respiratory tract infections were diagnosed. Data collected: gender, age, triage group, chronic diseases, vital signs, and PED-prescribed treatment (medications, dosages, methods of administration). Statistical analysis used SPSS 28.0, with significance at *p* < 0.05. Results. Data from 473 patients were analyzed, median age 3.5 years. Chronic diseases were present in 17.1% of children. 387 medications were prescribed, 47.5% being off-label. Off-label treatment was common for external otitis, acute laryngitis, and acute bronchitis (*p* < 0.001). There was incorrect administration of tobramycin with dexamethasone for otitis (*n* = 16, 100%) and inappropriate use of salbutamol inhalations by age (34.8%, *n* = 16). Some medications were given orally instead of injections (ondansetron *n* = 5, 62.5%; dexamethasone *n* = 82, 98.7%) or intranasally instead of intravenously (IV) (midazolam *n* = 7, 87.5%). IV adrenalin was prescribed for inhalations (*n* = 46). Younger children were more likely to receive off-label treatment (*p* < 0.001). Conclusion. Our study highlights the widespread issue of off-label and unlicensed drug prescribing in pediatric emergency care. Further research is necessary, because this reliance on off-label prescribing raises concerns about patient safety and compliance, especially given the limited clinical trials and therapeutic options available.

## 1. Introduction

The widespread off-label drug use in the pediatric population persists in both ambulatory and in-hospital settings despite collaborative efforts across multiple disciplines to increase scientific evidence and secure regulatory approvals for pediatric medications [1,2]. Many factors contribute to this phenomenon, including the absence of standardized prescribing information, a shortage of medications officially sanctioned for use in children, and insufficient clinical trials on account of ethical and logistic difficulties [3]. These factors collectively elevate the risk of exposing the pediatric population to drugs that lack comprehensive safety and efficacy data [4]. Off-label treatment is described as the use of licensed medication not according to the description in the product information [2], e.g., used for a disease that it is not approved to treat, administered in a way that is not described, or used in an inappropriate dose.

Off-label treatment in children is broadly researched and discussed in various studies. There is a tendency that mostly off-label or potentially off-label treatments are based on indication, a little less due to age, related to weight, or a combination of all [5]. It is worth mentioning that the youngest age groups of children are more likely to receive such treatment. In terms of diagnosis groups, it has been observed that respiratory diseases are often associated with treatment above the labeled instructions [6]. This underscores the substantial prevalence of off-label drug use in the pediatric ambulatory setting.

Recognizing that children are a very heterogeneous group with various physiologic development stages and other factors [2], it is crucial to acknowledge that anatomical and physiological changes within the pediatric population can significantly influence exposure to pharmacological substances. Consequently, careful dose adjustments become essential to limit any adverse effects. Unlike adults, where conventional dosage forms are widely accepted, the acceptability of dosage forms in pediatrics depends on individual characteristics such as age, competence, and developmental stage [7]. Together with ethical issues, it complicates the implementation of high-quality trials and underscores the necessity for relying on real-world data. The market size is also limited, and current incentives may not be enough in specific cases [2]. Challenges in developing pediatric medicines include high costs, a limited market, and methodological and ethical requirements [2,6]. Further clinical studies overcoming child-specific difficulties and systematic evaluation are needed to enhance pediatric pharmacotherapy.

In this study, we aimed to identify factors associated with off-label use of medication in children with respiratory tract infections in our pediatric emergency department (PED).

## 2. Materials and Methods

### 2.1. Study Design and Study Population

An exploratory retrospective single-center study was performed at the Lithuanian University of Health Sciences Kaunas Clinics Pediatric Emergency Department (PED). Case records of all patients aged 0–18 years referred to PED from 1 September to 1 October 2022, were analysed. The included month was randomly selected.

### 2.2. Data Collection

Demographic data (age, gender), triage group, according to Manchester Triage System (MTS) and all diseases (chronic among others), and final diagnosis were included into the study. Vital signs, such as respiratory rate (RR), heart rate (HR), temperature (T), and oxygen saturation (SpO2), were further analysed. According to MTS, patients were divided into groups 1–2 (resuscitation and emergency), triage group 3 (urgent), and 4 (nonurgent). Medication administered, their doses, and administration methods were collected. Patients were stratified based on whether they received an off-label medication, and further categorized into subgroups depending on whether they received one or two off-label medications. The annotations of medicines used in PED were also examined.

### 2.3. Definitions

Off-label treatment is using licensed medicines for indications that have not been approved by a national medicines regulatory authority [8].

Patient weight data were not documented in all collected hospital discharge reports, so we determined the correct dosage based on the child’s age.

### 2.4. Statistical Analysis

Data were collected using MS Excel and statistical analysis was conducted with SPSS 28.0 for Windows. Qualitative data were presented as counts (*n*) and percentages (%). We used the Shapiro–Wilk test to determine whether the data were normally distributed. Continuous variables were expressed as mean with standard deviation (SD) or median and interquartile range (IQR). Groups of children with or without off-label treatment were compared by an independent samples *t*-test if the data were normally distributed and Mann–Whitney U test for non-parametric data. The chi-square tests were applied to compare categorical variables and Pearson’s chi-square test was used to assess differences between samples. Logistic regression models were run to evaluate the strength of the association between the criteria, 95% confidence intervals (95% CI) for each variable were calculated. A value of *p* < 0.05 was considered significant. Data was presented in tables and figures.

### 2.5. Ethics

This study was conducted in accordance with the guidelines detailed in the Declaration of Helsinki and Good Clinical Practice Guidelines. Permission to conduct the study was obtained from a Regional Biomedical Research Ethics Committee (BE-2–27).

## 3. Results

### 3.1. General Characteristics of the Study Population

In total, 2633 patients were referred to PED during the study period. 473 children were diagnosed with respiratory diseases and were further analyzed. In total 202 were female (42.7%) (Table 1); the median age of all patients was 3.6 years (IQR 2.0–5.8). Medications were prescribed for 387 of them. The majority (*n* = 405) of patients were triaged as level 4 (not urgent), 64 patients in level 3 (urgent), and only four patients in as 1–2 (resuscitation and emergency). A total of 81 patients (17.1%) had chronic conditions, such as asthma, atopic dermatitis, allergic rhinitis, or developmental disorders. Additionally, 64 patients (13.5%) had visited their general practitioner (GP) before arriving at the PED. The majority of patients referred to PED complained of upper respiratory tract infection (*n* = 167), acute croup (with and without obstruction) (*n* = 72), external, middle, and internal otitis (*n* = 72), bronchitis (*n* = 40), and other respiratory tract diseases (Table 1).

### 3.2. Off-Label Medication

Out of a total of 203 (52.5%) medicines were administered in the appropriate method and the correct dose based on age, whereas 184 (47.5%) medications were given off-label. Among the total cohort of 473 patients, 125 (26.4%) received off-label medications during the study period. Of these, 66 patients (52.8%) were prescribed a single off-label drug, while 59 patients (47.2%) received two off-label medications (Table 2).

Predominantly, off-label treatment was administered to patients diagnosed with external otitis (72.2%), acute croup (93.9%), and bronchitis (32.3%) (*p* < 0.001). Our data revealed that the most common misuse involved medications being prescribed inappropriately. 76.1% of the off-label medicines were used in an unapproved route of administration, the rest were unapproved by age or indication (Table 3). The most commonly used off-label medications were ondansetron for relieving nausea and dexamethasone intravenous solutions, which were administered orally (*n* = 5, 62.5%, *n* = 82, 98.8% respectively). In children with acute croup, intravenous adrenaline was prescribed for inhalations (*n* = 46). Additionally, intranasal midazolam was administered instead of intravenous to seven children (prescribed before procedures). In otitis cases, 16 children (22.2% from all otitis cases) received tobramycin combined with dexamethasone eye drops. Salbutamol inhalations were utilized in 36.2% (*n* = 17) of children younger than four years old when diagnosed with obstructive bronchitis (Table 4).

The median age of patients receiving medications on-label was 5.3 years (4.7–5.9). Patients who were administered one medication off-label had an average age of 4.61 years (3.7–5.6), while those receiving two off-label medications were younger (2.98 (2.47–3.49)) (Table 4). Our observations indicated that younger children were more likely to receive off-label medications (ordinal regression estimate −0.17, 95% CI −0.257 to −0.084; *p* < 0.001). There was no significant difference in the prevalence of off-label drug usage between male and female patients or among triage groups (Table 5). Similarly, no significant variations were noted concerning the presence of chronic diseases or previous GP referrals and the administration of off-label treatment in the PED (Table 6).

## 4. Discussion

Off-label and unlicensed drug prescribing in the pediatric population is a pervasive and worrisome global concern. Our study focused on examining off-label prescriptions for children referring to our PED with respiratory tract infections. We observed that nearly half of the children received off-label medication, with a higher likelihood among younger patients.

The utilization of off-label or unlicensed medication is not prohibited [9]. In pediatric clinical practice, the lack of clinical trials due to specific methodological concerns, legal issues, and physiological, metabolic, and pharmacological peculiarities of this age group leads to a significant number of prescriptions involving off-label and unlicensed drugs. The absence of information on the drug label implies that the evidence required by regulatory authorities for its inclusion was either insufficient or not submitted for approval. A 2007 Delphi survey emphasized that contraindications specified in the summary of product characteristics should be strictly reserved for cases where there is clear evidence that the product should not be administered to children. Nevertheless, this restriction does not inherently inimply that the use of these drugs is inappropirate, as evidence-based decision-making can still benefit the patients [10]. For instance, the majority of medications prescribed in neonatal intensive units are off-label or unlicensed; yet these prescriptions are based on evidence practice. This situation arises due to deficiencies in the current licensing system [11]. Moreover, the risk of adverse reactions associated with off-label or unlicensed drugs is often compared to or lower than that of licensed drugs, serious adverse reactions are rarely observed [12].

The reported proportions of prescriptions for off-label and unlicensed medicines among children vary from 10 to 92% [13,14,15,16,17,18,19]. Our results did not differ, and analyzing only one month, we discovered that close to 50% of children with acute respiratory tract conditions are prescribed off-label medication in our PED. There are not a lot of studies examining PED prescriptions, however, considering the profile of the care in PED, taking into account its fast-paced environment, healthcare providers must make rapid yet informed decisions regarding treatment options. In most cases, it implies evidence-based guidelines and clinical experience. However, due to the urgent nature of many pediatric emergencies, off-label drug prescribing has become a common practice in PEDs. Nevertheless, the notable prevalence of off-label prescriptions in our environment can be attributed to the characteristics of our pediatric cases, with the majority being non-urgent and primarily outpatient in nature.

The impact of a child’s anatomy and physiology on drug formulation is significant. Several differences in the gastrointestinal tract between pediatric and adult populations affect drug absorption, distribution, metabolism, and elimination [7]. Due to higher metabolism, specific dosages should be adjusted, and in the majority of children are counted as per mass units of active compound per kg [7]. Despite specific calculations, many medications still lack appropriate formulations for the pediatric population due to the unique considerations of bioethics and legislation in this field. According to the European Medicines Agency (EMA), only approximately 40% of active substances are approved for pediatric use [20,21]. Additionally, medication adherence varies with age; for example, younger children, infants, and neonates may not be able to use tablets or capsules and some of the children can refuse medication due to specific taste [22]. Furthermore, the administration and adherence to further recommendations heavily rely on caregivers or healthcare professionals. In our settings, we noted specific administration variations from the primary formulation of the medication. Drugs such as ondansetron, dexamethasone, or midazolam were administered orally. This could be attributed to the fact that the conditions did not necessitate prolonged admission requiring venous access. Furthermore, venous access can be painful and stressful, making alternative administration routes more suitable for pediatric patients. Our results align with the research conducted by Taylor et al. [23]. The researchers revealed that midazolam, adrenaline, and ondansetron were predominantly prescribed off-label in most cases in PED. Lindell-Osuagwu et al. found out that 56% of the midazolam prescriptions were off-label [24]. Kaisto et al. observed 73% [14] of the cases receiving salbutamol treatment were off-label. In contrast, our findings indicate that 34.8% of patients were prescribed off-label salbutamol. The difference can be explained by the nature of the cases, departments, and study period.

Age is another highly important factor in off-label or unlicensed prescriptions. Various studies assert that there is a notable escalation in off-label drug utilization among children under the age of 2 years [25,26,27,28,29,30]. The prescriptions can be performed by trial because there is a shortage of therapeutic options in this age group. In our study, we noticed that younger children were administered off-label medication, and there was a trend of prescribing more off-label drugs to younger patients. Subsequent logistic regression analysis revealed a significant association between age and off-label prescriptions. Our data confirms that the lack of pediatric clinical trials and interest in performing clinical studies due to different concerns exposes children to excessive use of off-label medication, especially in the early stages of life. This can lead to adverse effects followed by low compliance. For sure, our study has its limitations due to sample size or strict collection period as well as specific conditions included. Moreover, we did not focus on the specific outcomes or adverse reactions. Nevertheless, it contributes additional knowledge to the pool of research on off-label drugs in pediatrics. Considering pediatric emergency, primary care, or in-hospital stay, more analysis should be performed to provide clear data on different medication groups in various conditions.

## 5. Conclusions

In conclusion, our study highlights the widespread issue of off-label and unlicensed drug prescribing in pediatric emergency care. Our findings, consistent with previous research, reveal a high prevalence of off-label drug use in pediatric emergencies, particularly among younger children. However, this reliance on off-label prescribing raises concerns about patient safety and compliance, especially given the limited clinical trials and therapeutic options available. Further research is necessary to fully understand the impact of off-label drug use on pediatric outcomes. Despite limitations, our study contributes to the existing knowledge on this topic and emphasizes the importance of ongoing research and monitoring in pediatric pharmacotherapy.

## Figures and Tables

**Table 1:** General characteristics.

Number of patients included, *n*		473
Median age, *year (IQR)*		3.6 (2.0–5.8)
Age groups, *n (%)*	≤28 days	2 (0.4)
	29 days–23 months	117 (24.7)
	2–11 years	333 (70.4)
	≥12 years	21 (4.4)
Gender	Female, *n (%)*	202 (42.7)
Triage group, *n (%)*	1–2	4 (0.8)
	3	64 (13.5)
	4	405 (85.6)
Chronic diseases, *n (%)*		81 (17.1)
GP referral, *n (%)*		64 (13.5)
Acute croup, *n (%)*		72 (15.2)
Asthma (with/without exacerbation), *n (%)*		10 (2.1)
Bronchitis, *n (%)*		40 (8.5)
Nasopharyngitis, *n (%)*		35 (7.4)
Acute otitis (externa, media), *n (%)*		72 (15.2)
Other diseases, *n (%)*		17 (3.6)
Pneumonia, *n (%)*		26 (5.5)
Tonsilitis, *n (%)*		34 (7.2)
Upper viral respiratory tract infection (e.g., pharyngitis), *n (%)*		167 (35.3)

*n*—number, %—percentage, IQR—interquartile range.

**Table 2:** Off-label data characteristics.

In total medications prescribed, *n*		387
Off-label medications, *n (%)*		184 (47.5)
Patients who received off-label medications, *n (%)*		125 (26.4)
Off-label treatment, *n (%)*	1 off-label medication	66 (52.8)
	2 off-label medications	59 (47.2)
Median age of patients receiving medications off-label, *year (IQR)*		3.4 (1.9–4.9)

*n*—number, %—percentage, IQR—interquartile range.

**Table 3:** Reasons for off-label administration of medications.

Medication	Total, *n*	Off-Label, *n (%)*	Reasons of Off-Label		
			Not Approved for Age, *n*	Indications Not Approved, *n*	Route Not Approved, *n*
Adrenalin	46	46 (100.0)	0	0	46
Cetirizine	1	1 (100.0)	1	0	0
Ciprofloxacin	1	1(100.0)	0	1	0
Dexamethasone	83	82 (98.8)	0	0	82
Fluticasone	1	1 (100.0)	1	0	0
Ketoprofen	2	1 (50.0)	0	1	0
Maxitrol (Dexamethasone, Neomycin and Polymyxin B)	3	3 (100.0)	0	3	0
Midazolam	8	7 (87.5)	0	0	7
Mometasone	5	1 (20.0)	1	0	0
Ondansetron	8	5 (62.5)	0	0	5
Salbutamol	47	17 (36.2)	17	0	0
Tobradex (Tobramycin and Dexamethasone)	16	16 (100.0)	0	16	0
Triprolidine and Pseudoephedrine	1	1 (100.0)	1	0	0
Vibrocil (Dimetindene and Phenylephrine)	2	2 (100.0)	2	0	0
*Total, n (%)*	224	184	23 (12.5)	21 (11.4)	140 (76.1)

*n*—number, %—percentage.

**Table 4:** Off-label medications administered in PEM by diagnosis.

	Total Cases, *n*	On-Label	1 Off-Label Medication	2 Off-Label Medications
Acute otitis externa, *n (%)*	18	5 (27.8)	12 (66.7)	1 (5.6)
Acute croup, *n*	60	3 (5.0)	17 (28.3)	40 (66.7)
Upper viral respiratory tract infections, *n*	31	20 (64.5)	8 (35.8)	3 (9.7)
Acute bronchitis, *n*	31	21 (67.7)	2 (6.5)	8 (25.8)
Acute otitis media, *n*	13	10 (76.9)	3 (23.1)	0 (0.0)

*n*—number, %—percentage.

**Table 5:** Off-label medication prescription in different patient groups.

		1 Off-Label Medication	2 Off-Label Medications	*p* Value
Gender, *n (%)*	Female	28 (25.9)	20 (18.5)	0.442
	Male	38 (24.5)	39 (25.2)	
Age groups, *n (%)*	29 days–23 months	13 (23.6)	20 (36.4)	0.017
	2–11 years	47 (24.4)	39 (20.2)	
	≥12 years	6 (40.0)	0 (0.0)	
Triage group, *n (%)*	1–2	0 (0.0)	0 (0.0)	0.584
	3	9 (20.0)	12 (26.7)	
	4	57 (26.4)	47 (21.8)	
Chronic diseases, *n (%)*		13 (28.9)	6 (13.3)	0.272
GP referral, *n (%)*		10 (15.2)	4 (6.8)	0.398

*n*—number, %—percentage, *p*—probability, GP—general practitioner.

**Table 6:** Logistic regression analysis of factors associated with off-label prescriptions.

Factors	OR		95% CI		*p* value
Age	−0.17		−0.257–−0.084		<0.001
Factors	B	S.E.	Wald	95% CI	*p* value
Age groups	−0.634	0.264	5.775	0.316–0.890	0.016
Gendrer	−0.177	0.261	0.460	0.503–1.397	0.497
Triage group	0.001	0.323	0.000	0.531–1.887	0.997
Chronic diseases	−0.440	0.358	1.516	0.319–1.298	0.218
GP referral	−0.343	0.367	0.872	0.346–1.457	0.350

OR—odds ratio, CI—confidence interval, B—unstandardized beta, S.E.—standard error, Wald—Wald test, GP—general practitioner.

## Data Availability

The data presented in this study are available on request from the corresponding author.
